# Cyclooxygenase-2 and von Willebrand factor—an immunohistochemical study of the equine foot with and without laminitis, post-mortem perfused with paraffin oil

**DOI:** 10.3389/fvets.2025.1673415

**Published:** 2025-12-15

**Authors:** Bianca A. Underberg, Elke Van der Vekens, Barbara Drews, Sabine Kaessmeyer

**Affiliations:** 1 Division of Clinical Radiology, Department of Clinical Veterinary Science, Vetsuisse Faculty, University of Bern, Bern, Switzerland; 2 Graduate School for Cellular and Biomedical Sciences, University of Bern, Bern, Switzerland; 3 Divison of Veterinary Anatomy, Department of Clinical Research and Veterinary Public Health, Vetsuisse Faculty, University of Bern, Bern, Switzerland

**Keywords:** hoof, inflammation, vascular endothelial cells, histology, immunohistochemistry, COX-2, vWF

## Abstract

**Objective:**

Equine laminitis is a complex and potentially fatal disease characterized by severe vascular and inflammatory alterations within the equine foot. This study aimed to develop immunohistochemistry (IHC) protocols for the detection of cyclooxygenase-2 (COX-2) and von Willebrand factor (vWF) in equine feet with and without laminitis, post-mortem perfused with paraffin oil.

**Materials and methods:**

A total of 12 equine forelimbs from 8 horses were utilized in this study, divided into two study cohorts: one with laminitis and the other as a non-laminitis control. To develop the IHC protocols thoroughly, the tissue samples were categorized into three groups: fresh, frozen-thawed, and frozen-thawed-perfused. IHC protocols for COX-2, a marker for inflammation, and vWF, a marker for vascular endothelial cells, were developed, respectively, optimized for use across these tissue groups. Samples from both study cohorts were processed and morphologically analyzed to assess tissue preservation and efficacy of immunostaining techniques.

**Results:**

Once the optimal paraffin embedding as well as combination of blocking reagents, blocking duration and antibodies had been empirically determined, COX-2 and vWF immunostaining were successful in all tissue groups. Reducing the embedding time in paraffin from 24 h to 2 h and positioning the samples at a 45° angle within the embedding cassette optimized the cutting results for microtome sectioning. Immunostaining specificity was good and results of COX-2 and vWF in both study cohorts were comparable and reliable between all three tissue groups. COX-2 was considerably elevated in the laminits cohort, predominantly in basal and parabasal cells, fibroblasts, and endothelial cells. vWF immunostaining effectively highlighted the vascular endothelial cells, revealing vascular compression and dilation, especially in the laminitis cohort.

**Conclusion/discussion:**

This study provides evidence that COX-2 and vWF can be reliably detected in equine lamellar hoof tissues, after undergoing freezing, thawing, and perfusion treatments. The established IHC protocols represent valuable diagnostic tools for studying the inflammatory and vascular changes associated with the equine foot. These methods can be applied to post-mortem cadaver models that have been frozen and perfused with paraffin oil, thus reducing the number of live animals required for research.

## 1 Introduction

Equine laminitis represents a critical and life-threatening condition, ranked as the second leading cause of mortality in the equine industry, surpassed only by colic (1). This has led to extensive global research efforts aimed at elucidating pathophysiology and developing effective interventions for this debilitating disease. In the past two decades, researchers have established various experimentally induced *in vivo* models of endocrine-, sepsis-related, and mechanical overload–induced laminitis (2, 3). These models are crucial for laminitis research as much of the pathological mechanism occurs prior to its clinical presentation, and it is therefore difficult to do research in naturally occurring laminitis cases. Although it is well known that changes of vasculature within the foot play a crucial role in the development of laminitis (4, 5). To date, the exact pathophysiological mechanism of this disease is not fully understood (2). It would therefore be beneficial to have a non-invasive method available to detect early clinical signs of laminitis.

The use of cross-sectional, three-dimensional imaging modalities such as computed tomography angiography (CTA) or magnetic resonance angiography (MRA) of the equine foot is currently state-of-the-art to show the vasculature *in vivo*. Until now, cross-sectional diagnostic imaging of this region has only been described using contrast agents, which are costly and pose a risk of side effects (6–8). To explore these vascular changes with high-field MRA, we have recently developed a vascular cadaver model. For that study, disarticulated equine distal cadaver limbs were perfused with oily perfusate via a roller pump system to simulate blood flow, enabling the acquisition of a high-resolution three-dimensional non-contrast-enhanced Time-Of-Flight MRA sequence that can be used to visualize vascular changes in the equine foot without the need for contrast agents (9).

Immunohistochemistry (IHC) would be a valuable diagnostic tool (10) to validate the laminitis caused changes observed by MRA. This is because IHC and histopathology are well established and indispensable techniques in laminitis research for identifying lamellar morphological changes and the weakening of the dermal-epidermal interface across various forms of laminitis (11).

By utilizing the antibodies anti-von Willebrand factor (vWF) for labeling vascular endothelial cells (12) and anti-cyclooxygenase-2 (COX-2), an enzyme indicative of inflammatory processes in the hoof tissue during laminitis (13), both the vascularization and inflammatory processes can be visualized. vWF is primarily produced by vascular endothelial cells and is stored in specialized secretory organelles called Weibel-Palade bodies (14). Upon vascular injury or cellular activation, vWF is released into the bloodstream, where it forms elongated multimeric structures that bind platelets and initiate clot formation (15). COX-2 typically exhibits low expression in most tissues but is rapidly and transiently upregulated in response to inflammatory stimuli across various cell types, including fibroblasts, endothelial cells, and leukocytes. Notably, the lamellar hoof tissue expression of COX-2 is considerably elevated in several forms of laminitis compared to other inflammatory mediators (13, 16, 17).

Due to species differences, the use of antibodies against vWF and COX-2 in equine tissue is challenging, as only a few commercially available antibodies have been shown to reliably cross-react with equine targets (18). In addition, the reproducibility of immunohistochemistry is markedly influenced by minor changes during tissue fixation and tissue processing before immunostaining (19).

With this methodological study, we addressed the challenge of reproducibly applying specific and sensitive IHC protocols for immunostaining COX-2 and vWF in frozen-thawed, oily perfused equine feet from horses with and without laminitis to visualize vascular and inflammatory changes by IHC.

## 2 Materials and methods

### 2.1 Study cohorts and tissue groups

Paired forelimbs were collected from horses between 1 and 26 years, euthanized in the equine hospital of the Vetsuisse Faculty, University of Bern between January 2023 and August 2024 for clinical reasons unrelated to this study. Euthanasia was performed via intravenous injection of pentobarbital (Esconarkon ad us. vet., Streuli Pharma AG, Uznach, Switzerland). The forelimbs of one horse were collected from a local abattoir. Ponies and draft horses were excluded. The included limbs were divided into a laminitis cohort, in which we expected COX-2 to be upregulated, and a non-laminitis control cohort. All owners of the horses submitted to the hospital signed an informed consent form permitting the use of tissues and images for research purposes.

The non-laminitis control cohort only included animals without a history of lameness or horses with an acute fracture or injury. Horses with systemic diseases or chronic unilateral lameness and < 3 years old were excluded. The inclusion criteria for the laminitis cohort consisted of a history of acute or chronic lameness, in combination with clinical examination and radiographic findings compatible with laminitis, such as capsular or phalangeal rotation and/ or sinking of the third phalanx.

A total of 46 forelimbs were collected from 23 horses. From 19 horses, one forelimb was randomly selected, and from the remaining 4 horses, both forelimbs were collected, making a total of 27 limbs used for the study. During the development of the IHC protocols, 15 limbs from 15 horses were lost due to processing artifacts during histological preparation. Therefore, 12 limbs from 8 horses could be evaluated and scored using the finalized protocols (Table 1).

**Table 1:** Distribution of tissue samples and regions assessed by anti-COX-2 and anti-vWF per tissue group and study cohort.

Study cohort	Antibody	Tissue groups			Total per cohort
		Fresh	Frozen-thawed	Frozen-thawed-perfused	
Control cohort		2 samples	1 sample	3 samples	6 samples
	COX-2	16 regions	8 regions	24 regions	48 regions
	vWF	8 regions	4 regions	12 regions	24 regions
Laminitis cohort		2 samples	1 sample	3 samples	6 samples
	COX-2	16 regions	8 regions	24 regions	48 regions
	vWF	8 regions	4 regions	12 regions	24 regions
Total per tissue group					
		4 samples	2 samples	6 samples	12 samples
	COX-2	32 regions	16 regions	48 regions	96 regions
	vWF	16 regions	8 regions	24 regions	48 regions

### 2.2 Sample collection – freezing thawing perfusion process

Prior to processing, all limbs were prepared as previously published (9, 20). In brief: after euthanasia, as soon as possible, maximally within 24 h, the limbs were disarticulated at the level of the middle carpal joint. All distal limbs were grossly cleaned and clipped, and orthogonal radiographs were performed to exclude limbs with major foot pathologies other than laminitis. After radiography, the distal limbs of both the laminitis and the non-laminitis control cohort were divided into the following tissue groups: (1) to be directly processed, referred to as “fresh” tissue group or (2) stored in a vacuum-sealed bag and frozen for at least 3 months at −20 °C, then thawed for 12 to 24 h at room temperature before being further processed (referred to as “frozen-thawed” tissue group) or (3) the “frozen-thawed-perfused” tissue group was treated as the frozen-thawed tissue group and then additionally perfused with paraffinum perliquidum (Huile de paraffin, Ideal Chimic SA, Carouge, Switzerland) during non-contrast-enhanced Time-Of-Flight MRA image acquisition, according to Underberg et al. (9), before processing (number of cohorts and tissue groups summarized in Table 1).

In the frozen-thawed-perfused tissue group, the contralateral forelimb was used in 2 horses from the non-laminitis control cohort and in 2 horses from the laminitis cohort, allowing for direct comparison between fresh and frozen-thawed-perfused tissues. One hoof tissue sample was evaluated per foot.

### 2.3 Dissection of the equine foot

Depending on the tissue groups, hoof dissections were performed either immediately after limb collection (fresh tissue group), after a freeze–thaw cycle (frozen-thawed tissue group) or after a freeze–thaw-perfusion cycle (frozen-thawed-perfused tissue group).

**Figure 1:**
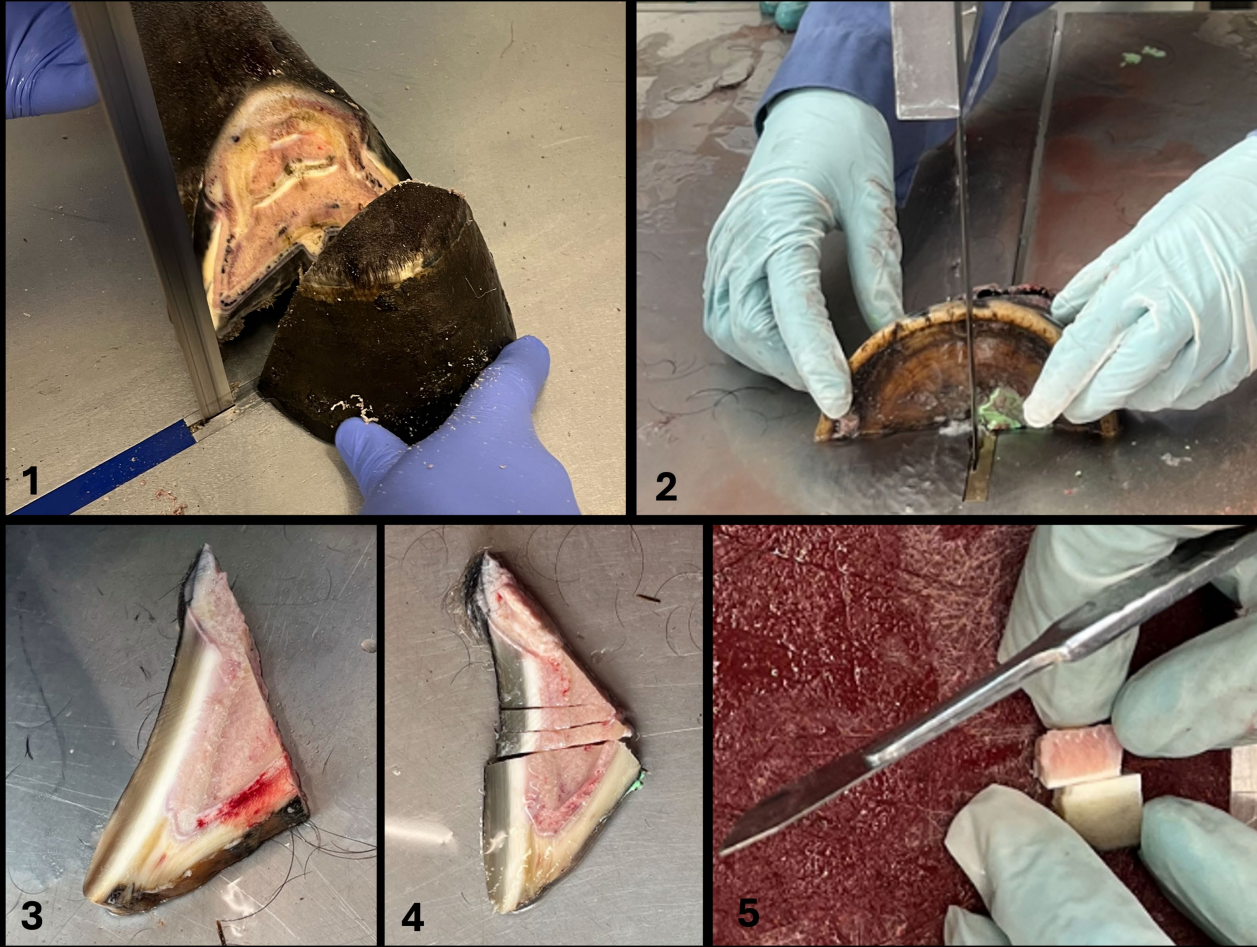
Cuts were made using a bandsaw for gross cuts and a scalpel for cutting smaller pieces according to Pollitt (21) (Figure 1, steps 1–5). For this purpose, the hoof was placed on the sole. The first saw cut was made in a mediolateral direction with a dorsal plane orientation, approximately 1 cm palmar to the most dorsal aspect of the coronary band to receive only the most dorsal tip of the foot (step 1). The dorsal hoof tissue was then placed on the cut surface (step 2). The second saw cut was made in a sagittal plane through the midline, followed by two other cuts, both abaxially to the sagittal cut, creating two approximately 1 cm thick wedge-shaped slices (step 3). Three transverse cuts were then made in the middle section of each wedge-shaped slice, perpendicular to the dorsal hoof wall, to obtain a total of four tissue blocks (step 4). They still included the dorsal hoof wall, the sublamellar dermis, and the dorsodistal aspect of P3. For the final two cuts, a scalpel was used to separate the sublamellar dermis from the parietal surface of P3 and to remove excess parietal horn tissue as much as possible to facilitate sectioning of the tissue with a microtome. Approximately 1 mm of epidermal horn remained on the samples (step 5).

Cuts were made using a bandsaw for gross cuts and a scalpel for cutting smaller pieces as previously described (21) (Figure 1, steps 1–5). For this purpose, the hoof was placed on the sole. The first saw cut was made in a mediolateral direction with a dorsal plane orientation, approximately 1 cm palmar to the most dorsal aspect of the coronary band to receive only the most dorsal tip of the foot (step 1). The dorsal hoof tissue was then placed on the cut surface (step 2). The second saw cut was made in a sagittal plane through the midline, followed by two other cuts, both abaxially to the sagittal cut, creating two approximately 1 cm thick wedge-shaped slices (step 3). Three transverse cuts were then made in the middle section of each wedge-shaped slice, perpendicular to the dorsal hoof wall, to obtain a total of four tissue blocks (step 4). They still included the dorsal hoof wall, the sublamellar dermis, and the dorsodistal aspect of P3. For the final two cuts, a scalpel was used to separate the sublamellar dermis from the parietal surface of P3 and to remove as much excess parietal horn tissue as possible to facilitate sectioning of the tissue with a microtome. Approximately 1 mm of epidermal horn remained on the samples (step 5).

### 2.4 Sample processing: embedding and microtome sectioning

**Figure 2:**
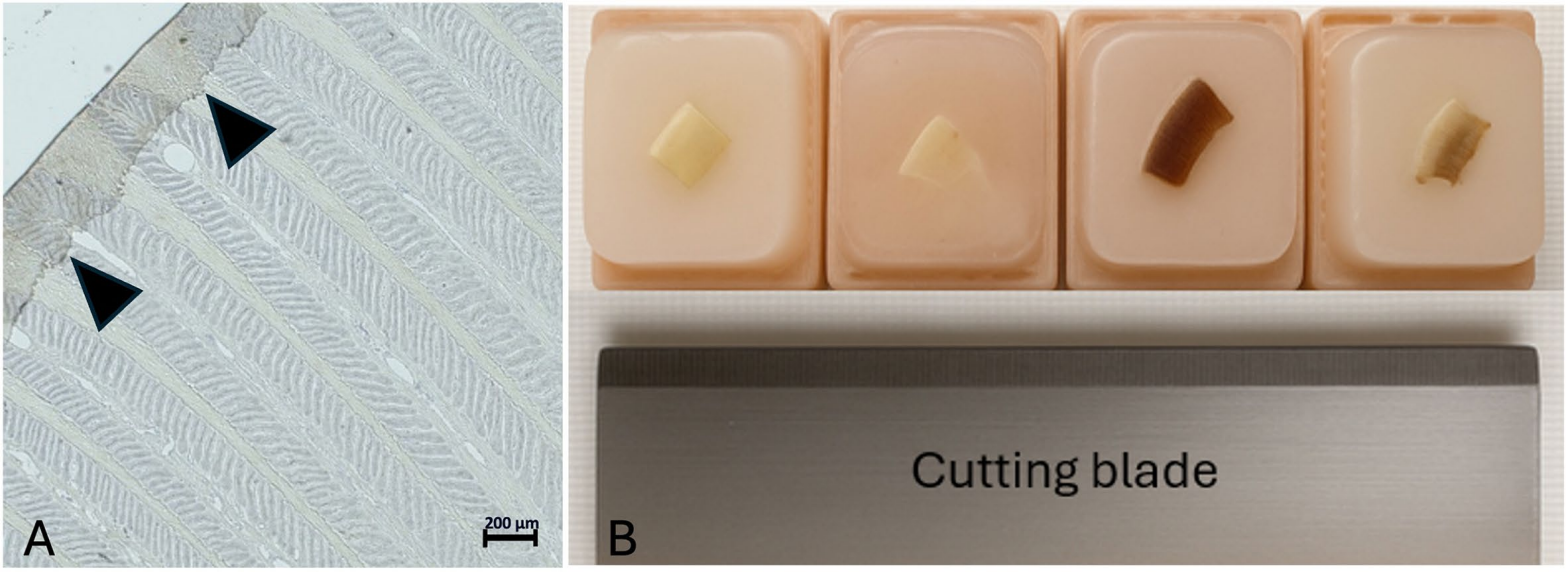
Tissue section showing rolled-up epidermal horn. **(A)** Arrowheads mark areas where the epidermal horn is rolled up. Light microscopy, scale bar = 200 μm. **(B)** Demonstration of the sample positioning within the paraffin block, so that the lamellae are oriented approximately at a 45° angle to the blade of the microtome.

Immediately after dissection, the remaining lamellar hoof tissue (10 length × 10 width × 8 height mm) was placed in embedding cassettes. The samples were fixed in 4% buffered formalin for 24 h at room temperature (RT) to preserve structural integrity. The samples were rinsed with tap water and washed in tris-buffered saline (TBS) for 15 min and were then dehydrated in ascending series of alcohol for 60 min respectively, 50 and 70% at 4 °C, then 90/100/100% at RT. They were cleared using Neo-Clear™ (CAS: 64741-65-7, Merck KGaA, Darmstadt, Germany, Distributor: Grogg Chemie, article number 1.09843.5000) for 2 h with a solution replacement after 1 h. Subsequently, the samples were infiltrated in liquid paraffin (Paraplast, Leica Biosystems Richmond, United States, article number 39601006) for 2 h at 60 °C (Embedding device Slee OT-02, Nieder-Olm, Germany) using a tissue processor. Samples were then placed in the embedding cassettes so that the lamellae were oriented approximately at a 45° angle to the cutting direction of the microtome blade (Figure 2). Sections of 2 μm thickness were cut with an Epredia™ HM 325 Rotary Microtome (Thermoscientific, Epredia, Microm International GmBH, Walldorf, Germany, Distributor Histocom) combined with a R35 disposable microtome blade Feather R35 (Biosystems article number. 81-0352-00). The sections for immunohistochemistry and histological evaluation were placed on Superfrost™ Plus Adhesion Microscope Slides (Epredia Adhesion Microscopic slides, New Erie Scientific LLC, Porthsmouth, United States, REF J1800AMNZ, Distributor, Biosystems).

### 2.5 Routine histological hematoxylin and eosin staining

Sections from each specimen were stained with hematoxylin and eosin (H&E). Briefly, after 24 to 48 h of drying at RT, microscope slides were placed in the incubator for 2 h at 36 °C and stained following a standardized protocol: the tissue sections were first immersed in three successive baths of Neo-Clear™, followed by graded alcohol series, starting with 100/90/80/70%. Next, the sections were placed in a filtered hematoxylin bath, washed in a 0.05% hydrochloric acid solution, and then rinsed with tap water to promote bluing. The sections were then immersed in 80% ethanol, followed by eosin-phloxin solution and 96% ethanol. Finally, two consecutive baths in 100% ethanol were performed, followed by three baths of Neo-Clear™. Finally, the tissue sections were mounted with cover slips using a mounting medium for microscopy (Neomount™, Merck, Darmstadt, Switzerland, article number: 109016).

### 2.6 Immunohistochemistry

#### 2.6.1 Antibodies

One polyclonal antibody, rabbit anti-vWF (Abcam Cambridge, United Kingdom, article number ab6994), and one monoclonal antibody, rabbit anti-COX-2 (Thermo Fisher Scientific, Massachusetts, United States, article number MA5-14568) were used (Table 2).

**Table 2:** Summary of primary antibodies, host species, supplier, catalogue number, dilution factor, incubation conditions, antigen retrieval methods, and control tissues used for immunohistochemistry.

Antibody	Type	Host	Supplier (Article No.)	Dilution	Incubation	Antigen retrieval	Negative control	Positive control
COX-2	Monoclonal	Rabbit	Thermo Fisher Scientific, Massachusetts, United States (MA5-14568)	1:100	24 h at 4 °C	Microwave heating in 10 mM citrate buffer: 8 min at 750 W, 10 min at 550 W	Sections of fresh lamellar hoof tissue of the non-laminitis control cohort	Sections of fresh lamellar hoof tissue from the laminitis cohort
vWF	Polyclonal	Rabbit	Abcam, Cambridge, United Kingdom (ab6994)	1:500	60 min at 4 °C	Microwave heating in 10 mM citrate buffer: 3 min at 750 W, 8 min at 550 W	Protocol without primary antibody	–
Both markers	–	–	–	–	–	–	Protocol without primary antibody	–

#### 2.6.2 IHC staining protocols

An IHC staining protocol for anti-COX2 was established within the framework of this study. The IHC staining protocol for anti-vWF used in this study is a modified version of a protocol developed in another study by our group that has not yet been published. Deparaffinization was performed in the same way for both antibodies, anti-vWF and anti-COX-2. The sections underwent deparaffinization in Xylol (5/5/3 min) and were subsequently rehydrated through a graded series of alcohol (100/100/90/80/70%). The subsequent epitope retrieval was achieved by a combination of heat and enzymatic processes. Therefore, all sections were heated in a microwave (Pelco BioWave Pro^®^; Ted Pella Inc., California, United States), while subjected in citrate buffer 10 mM, pH 6. vWF: 3 min at 750 W and 8 min at 550 W. COX-2: 8 min at 750 W and 10 min at 550 W. Followed by rinsing with TBS. Afterwards, slides were incubated with a protein block (component reagent of the Novolink™ Polymer Detection System, Leica Biosystems, Newcastle, United Kingdom) for 5 min and washed with TBS. Subsequently, slides were incubated with the primary antibodies, diluted in incubation buffer (50 mM pH 7,5 Tris + 140 mM NaCl + 5% Goat-Serum + 0.5% Casein + 0,1% NaN3): Anti-vWF (1:500): 60 min at 4 °C, anti-COX-2 (1:100): 24 h at 4 °C, then washed with TBS. All following steps were performed with further components of the Novolink™ Polymer Detection System and all slides were treated in the same way, regardless of the primary antibody applied. The sections were rinsed with peroxidase block for 5 min and rinsed with TBS. It was followed by incubation with the Post Primary which was supplemented with 5% Dog Serum for 30 min. After the washing step with TBS, Novolink™ polymer was applied for another 30 min. A last TBS rinsing followed, then antibody binding was visualized using the chromogen diaminobenzidine (DAB) working solution, which was applied for 5 min. The slides were then rinsed in water and counterstaining was performed with hematoxylin for 3 min. The slides were then rinsed under running tap water for 10 min. The sections were dehydrated through a graded series of alcohol (70/80/90/100/100%), before the clearing process in Xylol and mounted with mounting medium for microscopy (Neomount™, Merck, Darmstadt, Switzerland, article number: 109016) and covered with coverslips. For the negative controls, the COX-2 and vWF antibodies were omitted, and the sections were incubated with the dilution buffer only. Furthermore, the fresh lamellar hoof tissue of the non-laminitis control cohort was regarded as negative tissue control. Conversely, the fresh lamellar hoof tissue from the laminitis cohort was used as positive control for anti-COX-2.

### 2.7 Evaluation of tissue morphology and immunoreactivity

**Figure 3:**
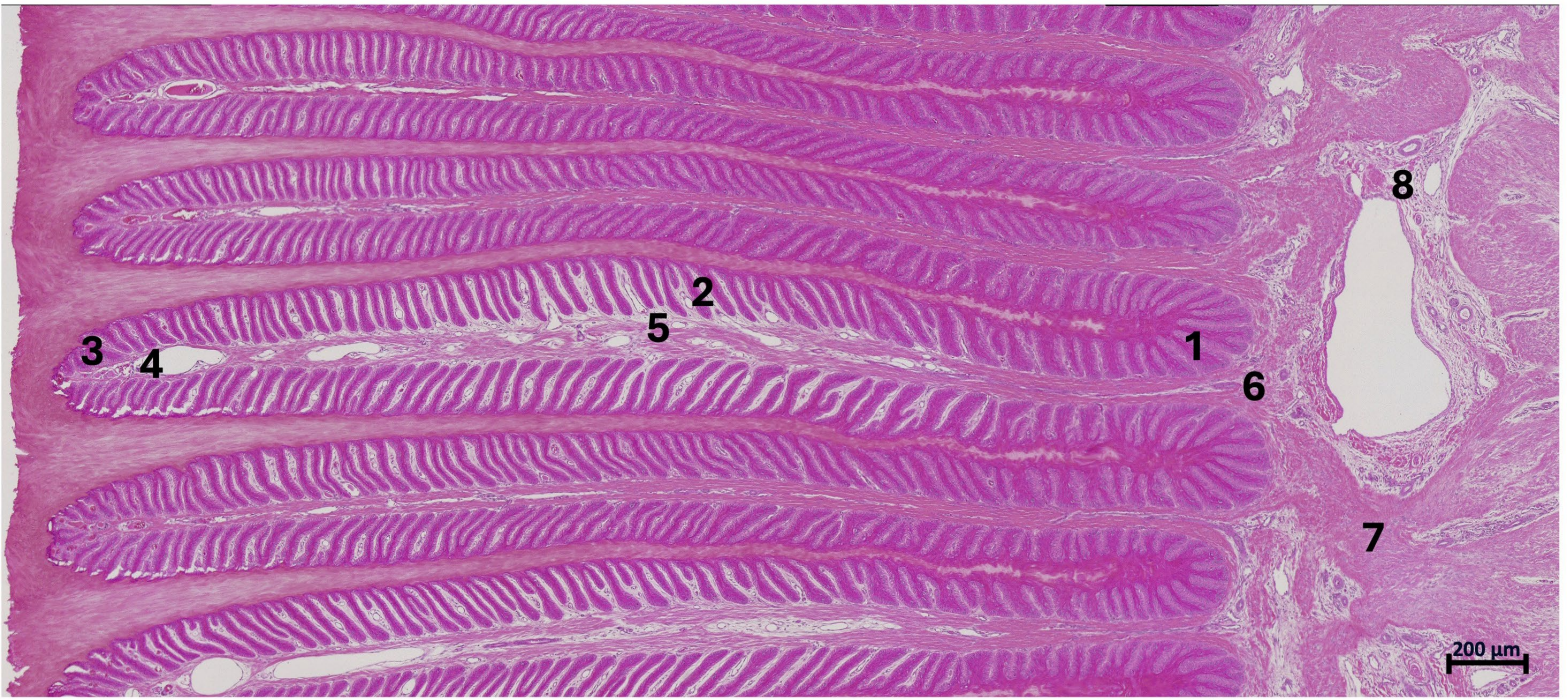
Overview image of a histologic section of fresh, non-laminitic lamellar hoof tissue stained with H&E. Left in the image is dorsal, right is palmar. The regions selected for histological analysis are base (1), middle (2), and tip (3) of the primary epidermal lamellae (PEL); tip (4), middle (5), and base (6) of the primary dermal lamellae (PDL); perivascular tissue (7) and blood vessels (8) within the sublamellar dermis. Light microscopy, scale bar = 200 μm.

A qualitative and semi-quantitative scoring system was chosen and adapted to subjectively score different parameters in different anatomical regions (13, 22). For this purpose, 8 anatomical regions were defined and evaluated in each histological sample as previously suggested by Patan-Zugaj (13). These included 3 regions of the primary epidermal lamellae (PEL): the tip (1), the middle (2) and the base (3); and 3 regions of the primary dermal lamellae (PDL): the tip (4), middle (5) and base (6). Furthermore, the perivascular dermal tissue (7) and the blood vessels (8), both located in the sublamellar dermis, were evaluated. Morphology and immunoreactivity were assessed by two researchers (BU, SK). Note: For each horse, one slide was analyzed, with all 8 tissue regions assessed for anti-COX-2 and only the 4 regions expected to contain blood vessels for anti-vWF. Evaluation was performed via light microscopy using a Zeiss Axio Imager with Apotome microscope (Carl Zeiss AG, Feldbach, Switzerland) at adequate magnifications (5x, 20x, 63x, respectively, 100x), according to the scored parameters. To demonstrate all regions, an overview image (Figure 3) was created (Keyence_VHX-5000, Frankfurt am Main, Germany).

#### 2.7.1 Routine histological H&E staining for morphological assessment

The H&E sections were used to examine the impact of the tissue pretreatment frozen-thawed and frozen-thawed-perfused on tissue architecture and cellular morphology in comparison to fresh. Tissue architecture was evaluated qualitatively according to form and shape of the hoof lamellae and overall cell morphology. The samples of each tissue group (fresh, frozen-thawed, frozen-thawed-perfused) were compared within the same study cohort (non-laminitis control and laminitis).

#### 2.7.2 COX-2 immunolocalization

The evaluation of immunoreactivity was performed in the whole histologic section of each sample. The cell types that exhibited COX-2 reactivity were assessed at 20x magnification and categorized as follows: basal epithelial cells, parabasal epithelial cells, fibroblasts, endothelial cells, intravascular leukocytes or “none” in case no cells were stained. To determine where the epitope is localized in the cells, 63x magnification was used and classified as: 1-nucleus membrane, 2-cytoplasm, and 3-both. Specificity was classified as low (1), moderate (2), or high (3). High specificity was characterized by a clear and well-defined demarcation between stained target cells and unstained cells. COX-2 expression was also semi-quantitatively evaluated based on the number of positively stained cells per image field at 20x magnification and the score was determined as follows: 0–none, 1-mild (1–50 cells), 2-moderate (50–100 cells), and 3-strong (>100 cells). The total score per sample was a minimum of 0 and a maximum of 24 (3 × 8, as 3 was the highest score achievable, and 8 regions were evaluated). Background staining was evaluated at 20x magnification and scored as: 0-none, 1-mild, 2-moderate, and 3-severe.

#### 2.7.3 vWF immunolocalization

The evaluation of immunoreactivity was performed in the whole histologic section of each sample. However, vWF labeling was only evaluated in regions 4, 5, 6, and 8, as regions 1, 2, 3, and 7 do not display blood vessels. The type of vWF-positive cells was evaluated at 20x magnification as described in 3.7.2. In addition, staining of the vascular lumen was assessed with no or yes. Immunostaining specificity for vWF was scored as low (1), moderate (2), or high (3), reflecting how selectively the immunostaining was limited to vascular endothelial cells. High specificity was defined by a clear and sharply delineated immunostaining of vascular endothelial cells. Lower specificity scores were assigned when immunostaining appeared diffuse or extended beyond the cell membranes of the endothelial cells; Background staining was evaluated at 20x magnification and scored as: 0- none, 1-mild, 2-moderate, and 3-severe. Semi-quantification was assed based on the number of stained endothelial cells (0: no stained cells; 1: less than half of the vascular endothelial cells, 2: more than half of the vascular endothelial cells, 3: all endothelial cells). The total semi-quantification score per sample (sum of all scores from 4 regions) was a minimum of 0 and a maximum of 12. In addition, the vascular lumen was characterized as compressed, not compressed, dilated, or not assessable, for example in case of lamellar separation.

## 3 Results

### 3.1 Study population and study groups

Twelve feet of 8 horses were evaluated and scored: 4 fresh feet (2 control, 2 laminitis), 2 frozen-thawed feet (1 control, 1 laminitis), 6 frozen-thawed-perfused feet (3 control, 3 laminitis). Reproducible COX-2 and vWF immunostaining protocols were established for all tissue groups: the fresh, frozen-thawed and frozen-thawed-perfused, and in both study cohorts: non-laminitis control and laminitis. To have a direct comparison, if possible, one forelimb per horse was used for the tissue group of fresh samples and the contralateral forelimb for processing after thawing and perfusion. This was possible in 4 out of 6 horses.

### 3.2 Optimized sample embedding and microtome sectioning

**Figure 4:**
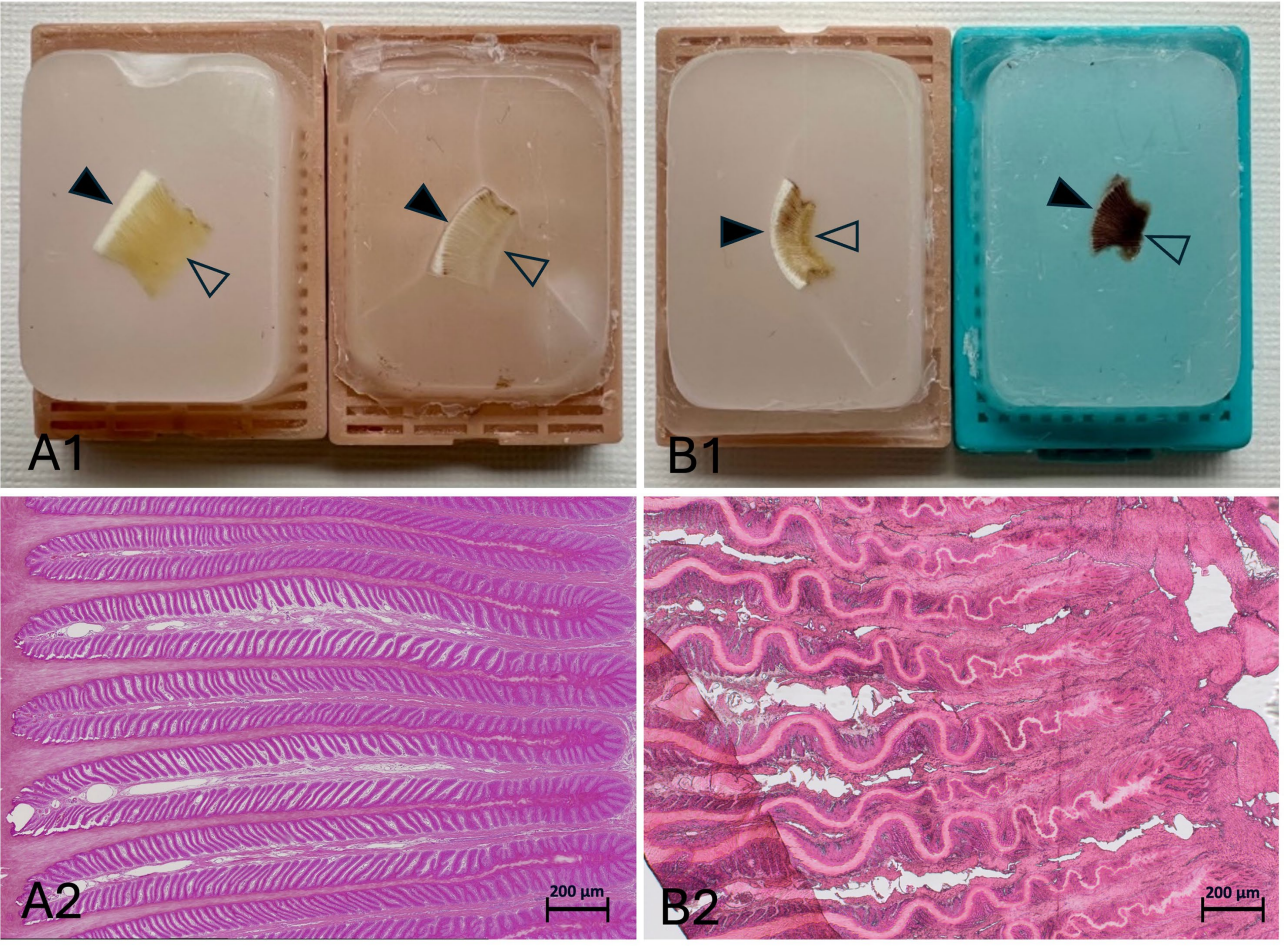
Impact of infiltration time on paraffin embedding quality. The solid arrowheads point toward the outer hoof wall, while the open arrowheads point toward the sublamellar dermis. The left side of the histological samples is facing toward the outer hoof wall; the right side is facing the sublamellar dermis. Two hours of paraffin infiltration resulted in well-maintained tissue structure, with no apparent shrinkage or discoloration **(A1,A2)**. After 24 h of infiltration, the tissue showed massive shrinkage and discoloration in both the paraffin block **(B1)** and the corresponding histological H&E section **(B2)**. **(A2,B2)** Light microscopy, scale bar = 200 μm.

During the first trials of fresh lamellar hoof tissue samples, 15 samples were lost due to massive tissue shrinkage and discoloration during the embedding process. Both the fresh and the pre-treated tissues shrank to half of their size and darkened during the embedding process for 24 h, which is the typical applied duration in our lab. Therefore, the first step, before continuing the embedding process with the remaining frozen-thawed and frozen-thawed-perfused tissue samples, was to process the tissues in a way that shrinkage could be avoided. Maintaining the tissue size and color was possible by adjusting the infiltration time in liquid paraffin to a maximum of 2 h and using automatic tissue processing machines. Examples of 2 h embedding time (left) vs. 24 h embedding time (right) are shown in Figure 4. Additionally, rolling of the epidermal part of the lamellae was noted during slice preparation. This was minimized by positioning the sample in the embedding cassette during paraffin embedding, so that the lamellae were oriented approximately at a 45° angle to the blade, ensuring the dermis faced the blade during microtomy shown in image (Figure 2B).

### 3.3 Routine H&E – morphological assessment of pretreatment groups and cohorts

**Figure 5:**
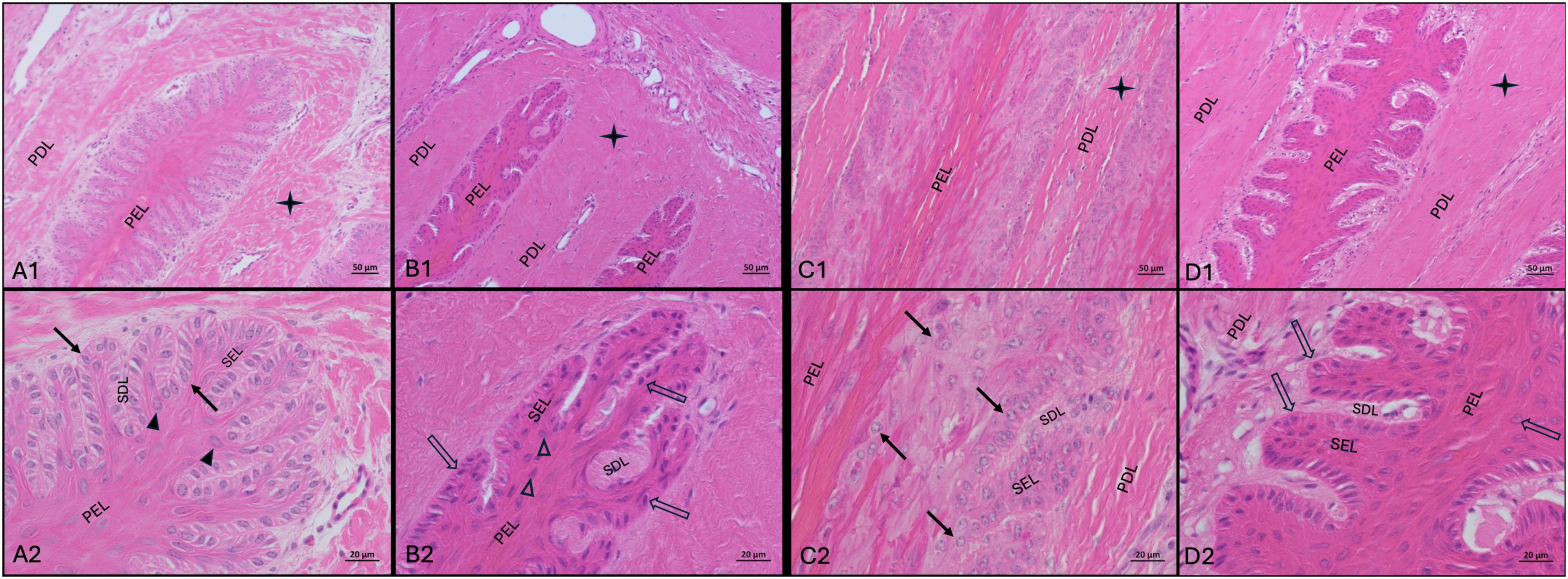
H&E-staining in lamellar hoof tissue from fresh **(A1,A2,C1,C2)** and frozen-thawed-perfused **(B1,B2,D1,D2)** samples (contralateral limbs). **(A1–B2)** Display sections from the non-laminitis control cohort and C1-D2 from the laminitis cohort. Images were taken in region 1 and region 6 (see material & methods, Section 3.7). PEL, primary epithelial lamella; PDL, primary dermal lamella; SEL, secondary epithelial lamella; SDL, secondary dermal lamella; black arrows: basal cell nuclei in fresh tissue; open arrows: basal cell nuclei in frozen-thawed-perfused tissue; solid arrowheads: parabasal cell nuclei in fresh tissue, open arrowheads: parabasal cell nuclei in frozen-thawed-perfused tissue. 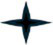 are indicating the connective tissues, Light microscopy, **(A1,B1,C1,D1)** scale bar = 50 μm; **(A2,B2,C2,D2)** scale bar = 20 μm.

The tissue integrity after freezing–thawing and perfusion processes was evaluated using H&E staining and light microscopic examination (Figure 5). Both the frozen-thawed and frozen-thawed-perfused hoof tissue samples showed moderate shrinkage of the hoof lamellar tissue. The connective tissue appeared more compact than in the fresh samples (Figures 5A1,B1,C1,D1), and the epithelial cells had a disturbed cellular shape. In particular, the basal epithelial cells and the parabasal cells were smaller, with a narrower cytoplasmic margin; their nuclei were darker and smaller, resulting in a lower visibility of the nucleoli and low differentiability from the cytoplasm (Figures 5A2,B2,C2,D2).

In lamellar hoof tissue samples from the non-laminitis control cohort, the tips of the secondary epithelial lamellae (SEL) were rounded, and the nuclei of the basal and parabasal cells were oval-shaped and oriented perpendicular to the axis of the SEL and located at the basal cell pole (Figures 5A1,A2). In the laminitis cohort, the tips of the SEL appeared elongated, and separation between the SEL and the secondary dermal lamella (SDL) was observed. The nuclei of most cells were rounded and had an irregular position within the cytoplasm (Figures 5B1,B2,D1,D2).

### 3.4 Evaluation of COX-2 and vWF-immunoreactivity

Once the optimal combination of blocking reagents, blocking duration and antibody had been empirically determined, COX-2 and vWF immunostaining was successful in all tissue groups. While COX-2 was assessable in all regions. vWF was not assessable in one region (Region 4) due to processing error (in the frozen-thawed-perfused group from the non-laminitis control cohort). In the laminitis cohort, 7 regions were not assessable due to separation of the lamellae, 3 in the fresh group (2x Region 4, 1x Region 5), 1 in the frozen-thawed group (1x Region 4), 3 in the frozen-thawed-perfused group (3x Region 4).

#### 3.4.1 COX-2 reactivity and distribution in the fresh tissue group of the non-laminitis and laminitis study cohort

**Figure 6:**
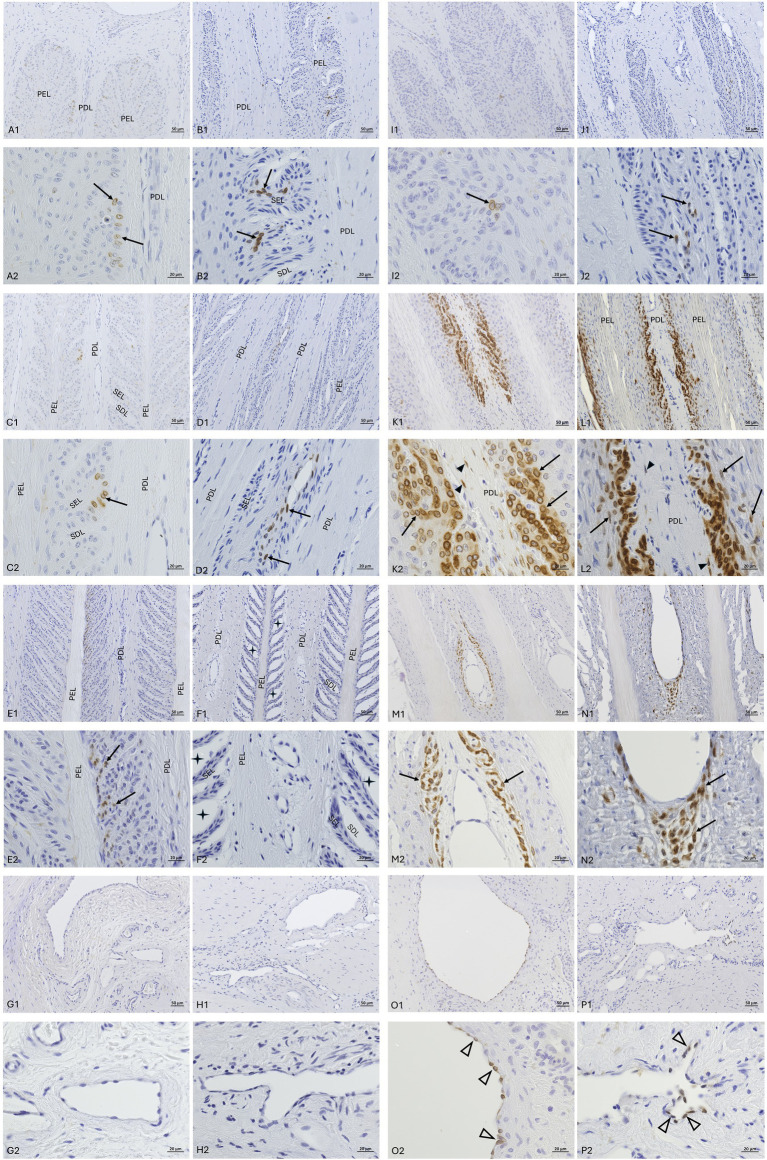
COX-2-IHC (DAB counterstaining) in lamellar hoof tissue from fresh **(A,C,E,G,I,K,M,O)** and frozen-thawed-perfused **(B,D,F,H,J,L,N,P)** samples. **(A1–F2)** Display sections from the non-laminitis control cohort, and **(I1–P2)** from the laminitis cohort. Images were taken in all selected regions (assignment to regions see Section 3.7). PEL, primary epithelial lamella; PDL, primary dermal lamella; SEL, secondary epithelial lamella; SDL, secondary dermal lamella; black arrows: COX-2 positive basal cells and parabasal cells; black arrowheads: COX-2 positive fibroblasts; open arrowheads: COX-2 positive endothelial cells; 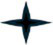: separations between the SEL and SDL; Light microscopy, **(A1–P1)** scale bar = 50 μm; **(A2–P2)** scale bar = 20 μm.

COX-2 immunolocalization was evaluated in fresh tissue samples from both the non-laminitis control and the laminitis cohorts (Figure 6). In the non-laminitis control cohort, the mean semi-quantification score was 1.5 (Range: 1–2) and, in the laminitis cohort, 12 (Range: 9–15). Positive COX-2 cells in the non-laminitis control cohort were only seen in regions 1 and 2. In the laminitis cohort, COX-2 immunostaining was detected in regions 1, 2, 3, 4, 5, and 8 (assignment to regions see Section 3.7). Furthermore, in the non-laminitis control cohort, only the epithelial cells exhibited immunostaining, while in the laminitis cohort, basal epithelial cells, parabasal epithelial cells, fibroblasts, and endothelial cells showed COX-2 expression. Of all cell types, the basal epithelial cells were the most frequently labeled cells.

#### 3.4.2 COX-2 reactivity and distribution in the frozen-thawed and frozen-thawed-perfused tissue groups of the laminitis study cohort

The distribution across the regions of COX-2 positive cells in the samples of the laminitis cohort was generally comparable between the two tissue groups (Figure 6); however, different quantities of stained cells were observed. The mean semi-quantitative expression score was 4 in the frozen-thawed tissue group and 5.3 in the frozen-thawed-perfused tissue group (Range 4–7).

Across all three tissue groups and study cohorts, region 2 showed, overall the most COX-2 positive cells (Figure 6).

#### 3.4.3 COX-2 immunostaining specificity and background immunostaining in the different tissue groups of the laminitis study cohort

**Figure 7:**
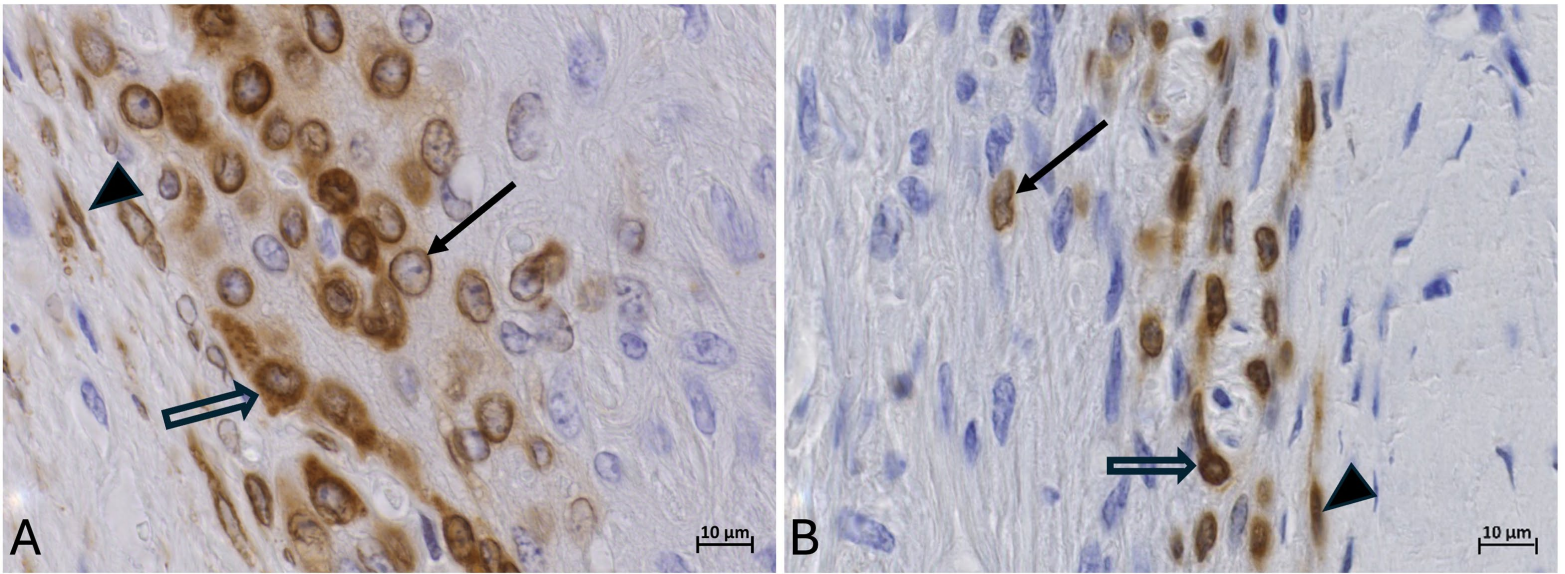
COX-2-IHC (DAB counterstaining) in the lamellar hoof tissue from the laminitis cohort in regions 2 and 5 (assignment to regions see Section 3.7). **(A)** Well-defined COX-2-immunostaining of the nuclear membrane (black arrow) and cytoplasm (open arrow) of keratinocytes in fresh lamellar hoof tissue sample. **(B)** COX-2 reactivity in a frozen-thawed-perfused lamellar hoof tissue sample of the same horse (contralateral limb) with reduced distinction between the cytoplasm and the nuclear membrane of lamellar epithelial cells (open arrow), COX-2 positive fibroblast (black arrow). Light microscopy, **(A,B)** scale bar = 10 μm.

For the immunostaining specificity of the labeled cell types, the fresh tissue group had a mean score of 2.55 (Range: 1–3), the frozen-thawed tissue group a mean score of 2.3 (Range: 2–3), and the frozen-thawed-perfused group a score of 2 (Range: 1–3). In the fresh tissue group, COX-2 labeling could be assigned well to specific cellular structures, mostly the nuclear membrane and the cell cytoplasm (Figure 7A). In rare cases either the nuclear membrane or the cytoplasm was stained (Figure 7A). This was observed in all COX-2 stained cell types, namely epithelial cells, fibroblasts, and endothelial cells.

In the frozen-thawed and the frozen-thawed-perfused tissue groups, it was not possible to assign the immunostaining to specific cell structures to a comparable extent as in fresh tissue, due to the described shrinkage of the tissue and altered cytoarchitecture (Figure 7B). However, the localization and distribution of the stained cells within the lamellar hoof tissue allowed a certain allocation to the target cell types.

For the background immunostaining, the fresh tissue group scored a mean background of 0.25 (Range: 0–1), the frozen-thawed group a mean score of 1 (Range: 0–2), and the frozen-thawed-perfused group a score of 0.17 (Range: 0–1).

#### 3.4.4 von Willebrand factor reactivity in the fresh tissue group of the non-laminitis and laminitis study cohort

**Figure 8:**
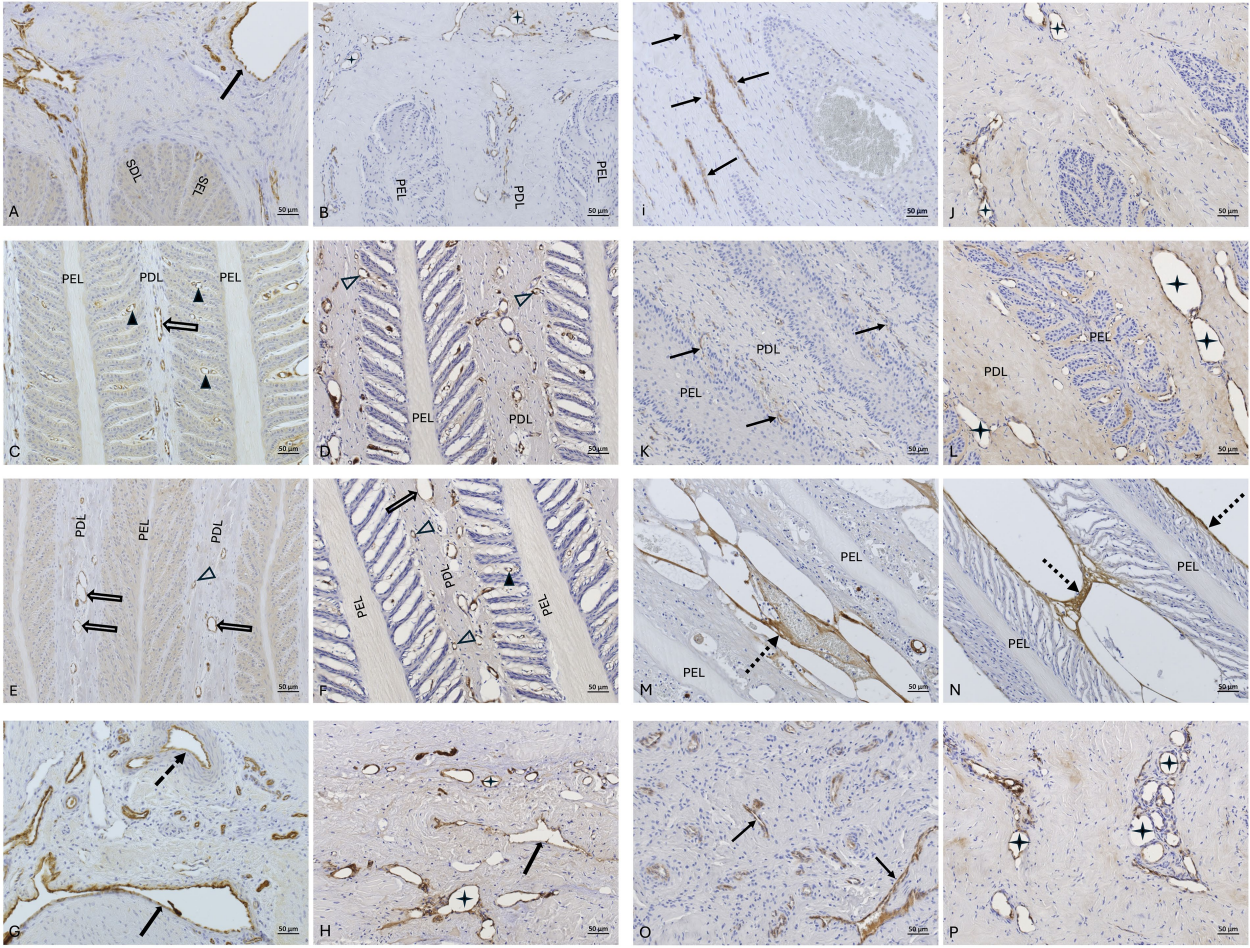
vWF-IHC (DAB counterstaining) in lamellar hoof tissue from fresh **(A,C,E,G,I,K,M,O)** and frozen-thawed-perfused **(B,D,F,H,J,L,N,P)** samples. **(A–H)** display sections from the non-laminitis control cohort, and **(I–P)** from the laminitis cohort. Images were taken in regions 4, 5, 6, and 8 (assignment to regions see Section 3.7). PEL, primary epithelial lamella; PDL, primary dermal lamella; SEL, secondary epithelial lamella; SDL, secondary dermal lamella; black arrows: vWF-positive sublamellar veins; dotted arrow: vWF-positive sublamellar artery; black arrowheads: vWF- lamellar capillaries; open arrowheads: vWF-positive lamellar arterioles/venules; open arrow: vWF-positive axial blood vessels within the lamellar dermis; 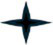 vWF-positive dilated blood vessels. Images M and N show small, dotted arrows pointing to unspecific immunostaining within the primary dermal lamellae (PDL), observed after lamellar separation. Note the background staining in the PDL in the image L. Light microscopy, scale bar = 50 μm.

vWF labeling of vascular endothelial cells was evaluated in regions 4, 5, 6, and 8 (assignment to regions see Section 3.7), both in the non-laminitis control cohort and in the laminitis cohort (Figures 8A–H). While in the non-laminitis control cohort, the semi-quantification score was 3, in the laminitis cohort, it was 2.7 (Range: 2.3–3). In some regions of the laminitis cohort samples, no clear immunostaining of endothelial cells could be identified because of lamellar separation.

#### 3.4.5 von Willebrand factor reactivity in the different tissue groups of the non-laminitis and laminitis study cohort

vWF immunostaining was successful in all samples of all tissue groups and both study cohorts (Figures 8A–P). The mean semi-quantification score for immunostaining of the vascular endothelial cells in the fresh tissue group was 2.8 (Range: 2.3–3), in the frozen-thawed group 3 (Range: 3), and in the frozen-thawed-perfused group 2.4 (Range: 1.7–3).

#### 3.4.6 von Willebrand factor immunostaining specificity and background staining in the different tissue groups of the non-laminitis and laminitis study cohort

**Figure 9:**
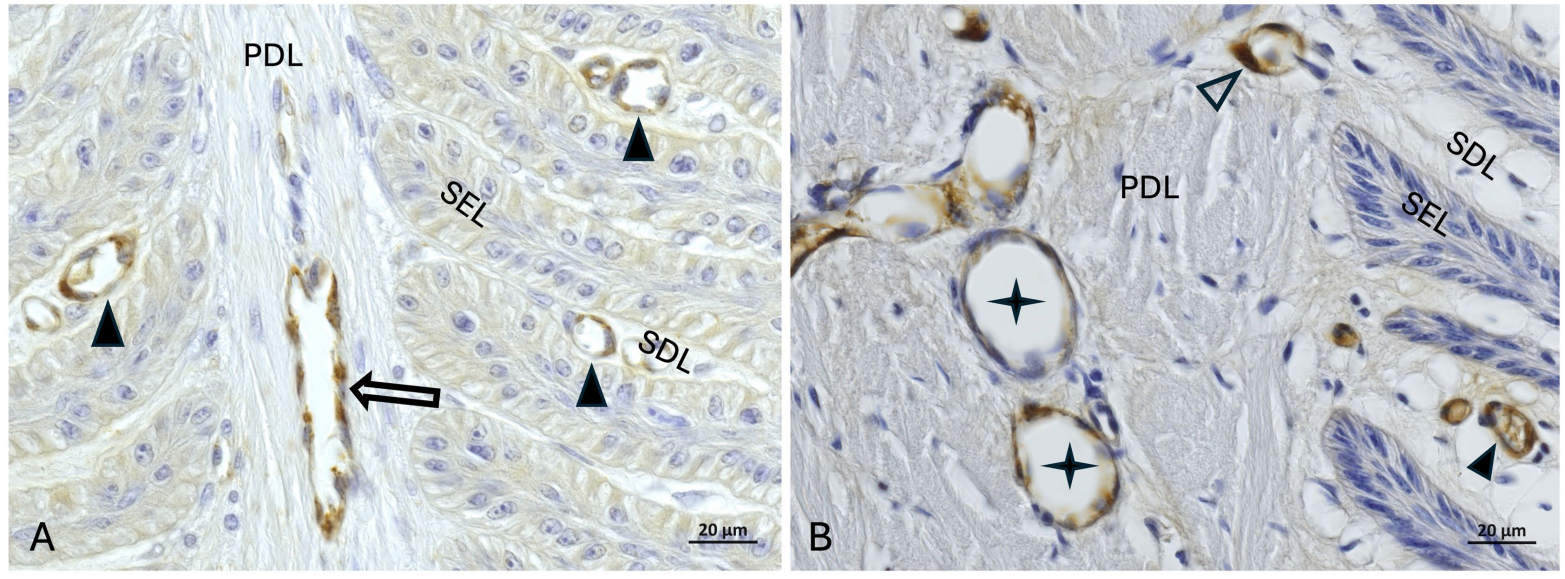
vWF-IHC (DAB counterstaining) in lamellar hoof tissue from fresh **(A)** and frozen-thawed-perfused **(B)** samples. Labeled vascular endothelial cells in regions 2 and 5 (assignment to regions see Section 3.7) in the non-laminitis control cohort. PEL, primary epithelial lamella; PDL, primary dermal lamella; SEL, secondary epithelial lamella; SDL, secondary dermal lamella; open arrow: vWF-positive axial blood vessels within the PDL, black arrowheads: vWF-positive capillaries in the SDL, open arrowheads: vWF-positive lamellar arterioles/ venules, 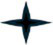 black stars: vWF-positive dilated blood vessels in the PDL. Light microscopy, **(A,B)** scale bar = 20 μm.

The mean score for immunostaining specificity of anti-vWF antibody in the non-laminitis control cohort was 3 in the fresh group, 3 in the frozen-thawed group, and 2.7 (Range: 2.3–3) in the frozen-thawed-perfused group. The mean score for immunostaining specificity of anti-vWF antibody in the laminitis cohort was 2.7 (Range: 2.3–3) in the fresh group, 3 in the frozen-thawed group, and 2.8 (Range: 2.6–3) in the frozen-thawed-perfused group (Figures 9A,B).

**Figure 10:**
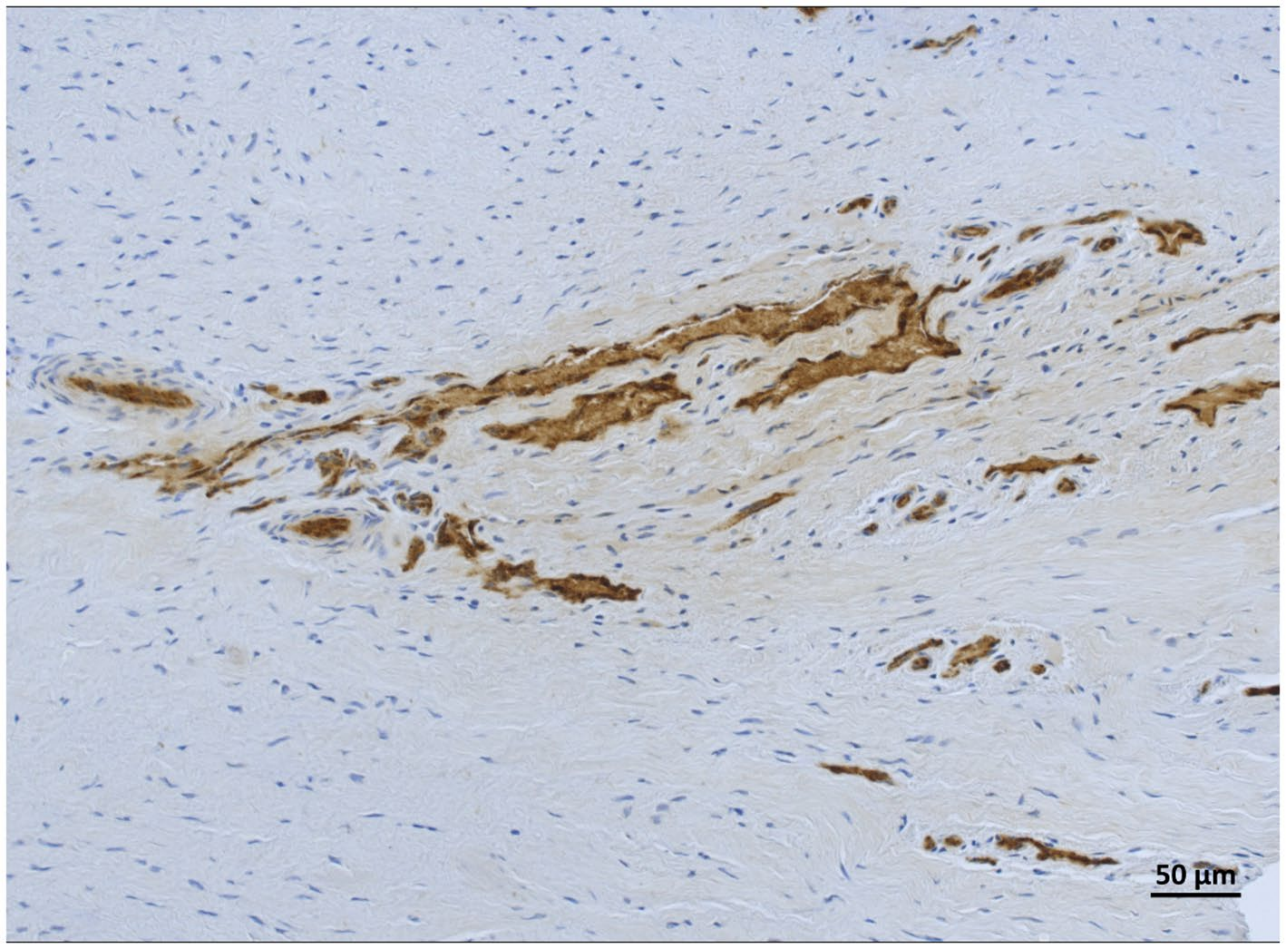
vWF-IHC showing stained vascular lumens in lamellar hoof tissue of a frozen-thawed sample in region 8 from the non-laminitis control cohort. Light microscopy, scale bar = 50 μm.

In the non-laminitis control cohort, immunostaining of the vascular lumen was present in 25% (2/8) of regions in the fresh group, 50% (2/4) in the frozen-thawed group, and 45% (5/11) in the frozen-thawed-perfused group. In the laminitis cohort, 20% (1/5) of regions showed immunostaining of the vascular lumen in the fresh group, 100% (3/3) in the frozen-thawed group, and 22% (2/9) in the frozen-thawed-perfused group (Figure 10).

**Figure 11:**
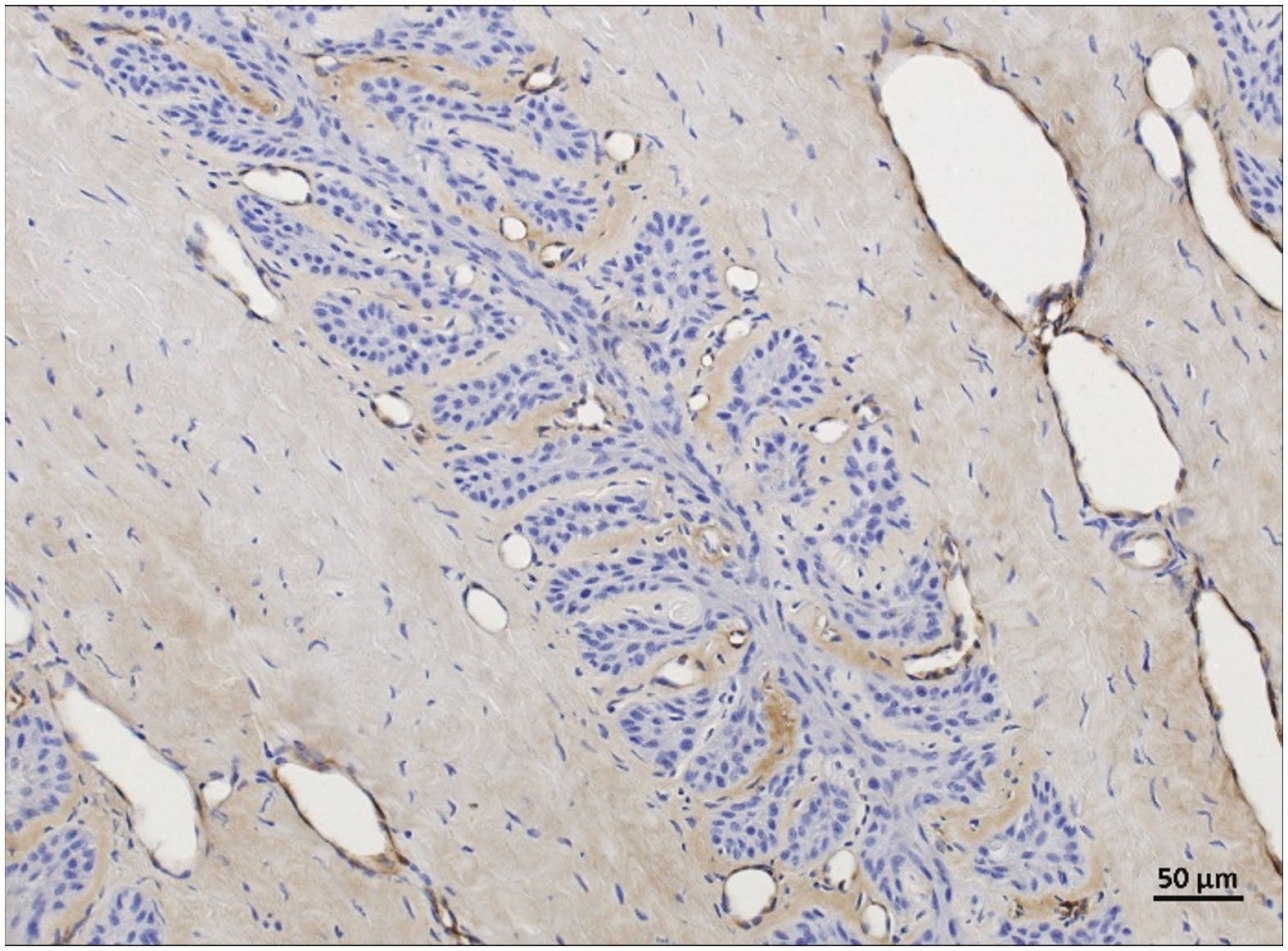
vWF-IHC showing non-specific background staining in frozen-thawed-perfused lamellar hoof tissue (region 2 and 5) of the laminitis cohort. Light microscopy, scale bar = 50 μm.

For background staining, all 24 regions were assessable in the non-laminitis control cohort as well as in the laminitis cohort. In the non-laminitis control cohort, the fresh group had a mean score of 1.9 (Range: 1.8–2), the frozen-thawed group had a mean score of 0.5, and the frozen-thawed-perfused group a score of 1.08 (Range: 0–3). In the laminitis cohort, the fresh group had a mean score of 0.87 (Range: 0–1.8), the frozen-thawed group had a mean score of 0.75, and the frozen-thawed-perfused group had a score of 1 (Range:0–1.8) (Figure 11).

#### 3.4.7 Compression of vascular lumen in the different tissue groups of the non-laminitis and laminitis study cohort

**Figure 12:**
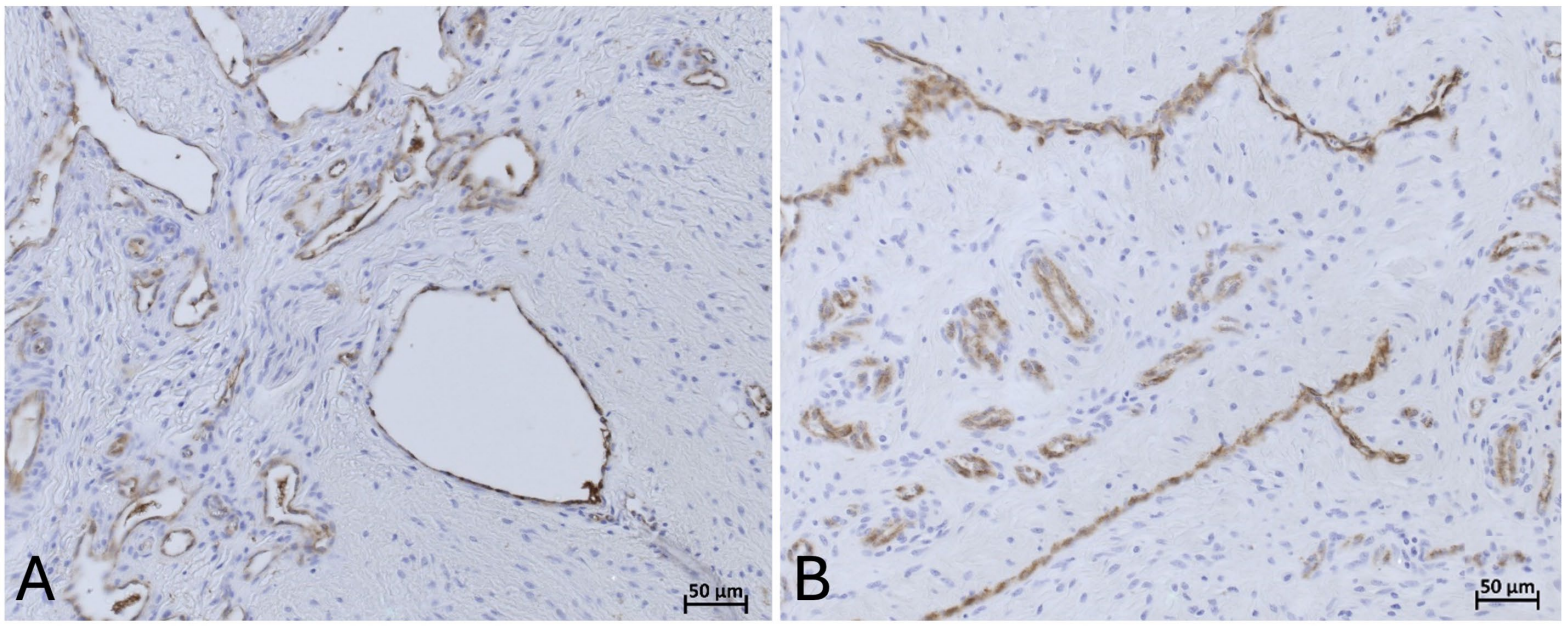
**(A)** vWF positive immunostaining (DAB counterstaining) highlights endothelial cells lining an open vascular lumen in a lamellar hoof tissue sample of a fresh non-laminits control cohort in region 8. **(B)** vWF positive IHC staining highlights endothelial cells lining a compressed vascular lumen in a fresh lamellar hoof tissue sample of the laminitis cohort in region 8. Light microscopy, **(A,B)** scale bar = 50 μm.

Across all tissue groups of the non-laminitis control cohort, 4% (1 of 23) showed compressed blood vessels, 21% (5 of 23) showed dilated blood vessels and 73% (17 of 23) blood vessels were not compressed; Across all tissue groups of the laminitis cohort, 59% of all assessable regions (10 of 17) showed compressed blood vessels, 24% showed (4 of 17) dilated vessels and 18% (3 of 17) blood vessels were not compressed. When comparing the different tissue groups unrelated to study cohorts, from the fresh samples, 31% (4 of 13) showed compressed blood vessels, 69% (9 of 13) showed no compression of blood vessels, and no vascular dilation was observed. In the frozen-thawed group, 28% (2 of 7) blood vessels were compressed, and 71% (5 of 7) were not compressed, no vascular dilation was observed. In the frozen-thawed-perfused group, 25% (5 of 20) showed compression of blood vessels, 30% (6 of 20) showed no compression of blood vessels, but 45% (9 of 20) showed dilated blood vessels (Figure 12).

In the frozen-thawed-perfused samples, some blood vessels exhibited loss of the perivascular connective tissue integrity, with no discernible pattern to specific regions. These observations were confirmed by comparing the fresh lamellar hoof tissue samples from the same horse but using the contralateral forelimb, where the tissue integrity was preserved.

## 4 Discussion

There is only a limited number of IHC staining protocols published for equine tissue in general (18, 23, 24). No IHC protocol has been described for use in hoof tissue after prior pre-treatment, such as freezing and thawing or perfusion with paraffinum perliquidum (paraffine oil). In this study, we have successfully and reproducibly established an IHC protocol for the immunostaining of COX-2. For the immunostaining of vWF, we modified and applied a protocol already established in the group (Drews et al., unpublished data). We performed the immunostaining on thawed cadaver feet perfused with oily perfusate of horses with and without laminitis. We were able to visualize COX-2 expression, indicative of inflammation, and vascular changes by labeling vascular endothelial cells with anti-vWF in laminitic hoof tissues.

Unlike ELISA or PCR, which only confirm the presence or concentration of a marker regardless of its localization, IHC staining allows for precise localization of target antigens, enabling researchers to determine exactly where the marker is expressed in the tissue (25). This capacity of IHC for specific antigen localization within a tissue also allows comparison with state-of-the-art post-mortem angiographies using CTA or MRA (9, 20) allowing future correlations between histological findings and structural pathology. The results of this study provide valuable information on the histologic methodology and treatment of equine lamellar hoof tissue. They serve as a comparative basis for the accurate interpretation and evaluation of post-mortem angiographies performed with thawed and oily-perfused equine distal limb cadaver models.

### 4.1 Hurdles in preparing hoof lamellar tissue for histological examination

Several challenges were encountered during the processing of equine lamellar hoof tissue, with one of the most serious being the tissue handling and processing prior to the embedding procedure.

Infiltration within liquid paraffin led to severe tissue shrinkage and discoloration, affecting all tissue groups. These artifacts, after the embedding process in liquid paraffin, likely resulted from prolonged exposure to high temperatures and dehydrating agents (26, 27). By shortening the liquid paraffin infiltration time to 2 h and utilizing an automated embedding system, these effects were successfully minimized (kind advice from Chris Pollitt through personal communication), as also described by Grizzle et al. (27).

Sectioning of hoof tissue is technically demanding, due to the complex hoof structure, which is composed of tissues with various densities. Particularly, the interface between the rigid keratinized epidermal and the softer dermal components makes cutting of histological sections difficult (28, 29). Different suggestions regarding preferred sectioning methods have been described, such as aligning the cutting blade parallel to the lamellae, sectioning the tissue oriented perpendicular to the lamellae or rather using an oblique orientation to the lamellae (28, 29). In our study, the oblique orientation yielded the best results, which is also recommended by Peters (29). We hypothesize that the oblique orientation showed best results as the cutting blade does not encounter the full cross-sectional surface of the paraffin-embedded hoof tissue. Instead, it first contacted the softer dermal edge which may have reduced resistance during sectioning and minimized pressure and tissue deformation.

### 4.2 Tissue shrinkage in pre-treated lamellar tissue resulting from the freeze–thaw process

Histological artifacts resulting from the freeze–thaw process were observed in the frozen-thawed and frozen-thawed perfused tissue groups. Specifically, the connective tissue appeared more compact than in the fresh tissue samples and the epithelial cells had an altered morphology. Cell alterations resulting from the freeze–thaw process are mainly caused by shifts in water between the intracellular and extracellular spaces, as the tissue temperature changes. When extracellular water freezes, it creates an osmotic gradient that draws water out of the cells, leading to increased intracellular solute concentrations. This results in cellular shrinkage and disruption of intercellular junctions (30). When the tissue is thawed, water re-enters the cells, which can cause swelling and even rupture of already damaged cells. These effects are thought to underlie much of the structural alteration observed in thawed tissue, rather than mechanical damage from ice crystal formation alone (31). After thawing, we quickly fixed the tissue to avoid further shrinkage. It also preserved the architecture of the tissue as far as possible.

Changes in chromatin structure are consistent with known freeze–thaw artifacts, which commonly involve nuclear condensation and enhanced chromatin staining (hyperchromasia) (29). This is compatible with our study showing darker and smaller nuclei resulting in a lower visibility of the nucleoli and low differentiability from the cytoplasm.

### 4.3 Specific COX-2 and vWF immunostaining was possible in all tissue groups and study cohorts

For optimal IHC results, tissue samples should be collected as fresh as possible, to minimize autolytic degradation. Autolysis can lead to the loss of enzymes, protein denaturation, and structural deterioration. It can therefore alter antigen conformation and impair immunoreactivity, which may obscure or distort the true pathologic state and must therefore be carefully considered when interpreting histologic findings in tissues (31, 32).

### 4.4 General and group-specific COX-2 immunostaining patterns

#### 4.4.1 Methodological considerations

Between the different tissue groups, the overall results of COX-2 immunostaining were comparable. However, due to the morphological alterations caused by the freeze–thaw process, including shrinkage of lamellar basal cells of the secondary lamellae—it was not always possible to assign the immunostaining precisely to specific cell types. In addition, immunostaining specificity was less clearly demarcated in tissues that underwent freeze–thaw cycles compared to freshly processed samples. Nevertheless, based on our observations, we hypothesize that the mid-region of the PDL (Region 2/5) is the most affected area, as COX-2 expression repeatedly showed the highest intensity in this region across all tissue groups of the laminitic cohort. Further investigation is required to confirm this finding.

#### 4.4.2 Pathophysiological interpretation

COX-2 plays a central role in inflammatory processes and is of major importance in both human and equine medicine, as it is a target enzyme for non-steroidal anti-inflammatory drugs (16, 33). Laminitis is recognized as an inflammatory condition, with studies-both those involving experimentally induced and spontaneous cases of laminitis-demonstrating increased COX-2 expression in the lamellae (13, 16, 34–39). Other studies have shown that cryotherapy can specifically reduce COX-2 expression compared to other inflammatory enzymes in horses with laminitis (16, 40).

The COX-2 immunostaining results of our study support these previous observations: increased COX-2 expression in laminitic samples reflected the inflammatory response. Elevated COX-2 expression was predominantly observed in lamellar epithelial basal cells, followed by parabasal cells, fibroblasts, and endothelial cells, consistent with findings of Patan-Zugaj (13). Notably, inflammatory activity was not restricted to epithelial cells but also affected blood vessels, as indicated by COX-2 expression in vascular endothelial cells. This may represent an early indicator of vascular involvement in laminitis and further supports the hypothesis that blood vessels play a key role in the disease process (4, 5, 41, 42).

In samples from the non-laminitis control cohort, mild COX-2 immunostaining was observed, aligning with previous studies describing low expression of COX-2 in non-diseased tissues (13). This expression may reflect systemic inflammation associated with each horse’s primary cause of death. Three control horses had been euthanized due to severe musculoskeletal trauma, while one horse had been presented with acute ataxia caused by neoplasia of the second cervical vertebra (C2) (histopathologically confirmed lymphoma). The latter showed the highest COX-2 prevalence. Interestingly, in approximately 60% of human non-Hodgkin’s lymphoma cases, increased COX-2 expression has also been documented (43). To our knowledge, no such investigation has yet been conducted in horses.

### 4.5 General and group-specific vWF immunostaining patterns

#### 4.5.1 Methodological considerations

To visualize endothelial cells, we applied an immunohistochemical (IHC) protocol adapted from a previous comparative study of CD31 and vWF. In that study, vWF showed superior immunostaining quality and reproducibility for endothelial cells in lamellar hoof tissue (Drews et al., unpublished data). Consistent with these findings, the protocol used here provided reliable visualization of vascular endothelial cells and blood vessels across all sample types—including fresh, frozen-thawed, and frozen-thawed-perfused tissues.

The observation of dilated, oily-perfused blood vessels corresponds with results from other post-mortem perfusion studies, where vascular dilatation has been reported following perfusion angiography (44). In addition, disruption of perivascular connective tissue integrity—referred to in other studies as “perivascular courts”—was occasionally observed, consistent with known artifacts of perfusion procedures, including perfusion fixation (31, 45). These perfusion-related mechanical and osmotic effects should be carefully considered when interpreting laminitic tissue samples that have undergone post-mortem perfusion with paraffin oil or when correlating histological findings with MRA imaging data (9).

#### 4.5.2 Pathophysiological interpretation

In the laminitis cohort, compression of the vascular lumen was observed in 59% of samples across all tissue groups, whereas none of the control samples (non-laminitis) exhibited this feature, except for one frozen-thawed-perfused sample. This particular tissue originated from the horse with the C2 lymphoma, which also showed increased COX-2 expression. The mixed pattern of dilated and compressed blood vessels in that case may therefore reflect systemic inflammatory effects associated with the neoplastic condition.

These findings support the interpretation of vascular compression as a morphological hallmark of laminitis, consistent with previous studies (8, 46–49). Our results therefore strengthen the hypothesis that vascular alterations and impaired blood circulation are key components of the pathophysiology of laminitis.

Furthermore, the combination of increased COX-2 expression and vascular compression in the lymphoma case, despite the absence of clinical signs of laminitis, may indicate an early or subclinical stage of the disease. This observation highlights the potential diagnostic value of endothelial and inflammatory markers in identifying early vascular involvement in laminitis.

## 5 Limitations

The loss of samples during method development reduced the number of specimens analyzed, which should not be underestimated. Additionally, the semi-quantitative evaluation of COX-2 and vWF expression could have been influenced by subjective assessments. The presence of vascular dilation in parallel to vascular compression in the entire laminitis cohort of the frozen-thawed-perfused tissue group raises concerns that this iatrogenic (artificial) dilation of possibly previously compressed vessels limits the diagnostic interpretation. While COX-2 and vWF proved to be valuable and reliable markers for identifying inflammatory and vascular alterations in lamellar tissues, they alone cannot fully capture the inflammatory and vascular complexity of laminitis. However, for the purpose of this methodological study, COX-2 and vWF were selected to establish and validate reproducible immunohistochemical protocols, whose results were suitable for comparison with state-of-the-art post-mortem MRA findings. Future investigations could build on this study by incorporating additional laminitis-relevant markers to achieve a more comprehensive characterization of disease-associated processes.

## 6 Conclusion

With this methodological study, we addressed the challenge of reproducibly applying specific and sensitive IHC protocols for immunostaining COX-2 and vWF in thawed, oily perfused equine feet from horses with and without laminitis to visualize inflammatory and vascular changes by IHC. The labeling of inflamed tissue as well as blood vessels can be used as a reference for diagnostic imaging approaches using a frozen-thawed cadaver model.

## Data Availability

The original contributions presented in the study are included in the article/supplementary material, further inquiries can be directed to the corresponding author.
